# Action Intentions Reactivate Representations of Task-Relevant Cognitive Cues

**DOI:** 10.1523/ENEURO.0041-25.2025

**Published:** 2025-06-18

**Authors:** Nina Lee, Lin Lawrence Guo, Adrian Nestor, Matthias Niemeier

**Affiliations:** ^1^Department of Psychology at Scarborough, University of Toronto, Scarborough, Ontario M1C1A4, Canada; ^2^Centre for Vision Research, York University, Toronto, Ontario M4N3M6, Canada

**Keywords:** beta band, grasping, memory, reaching, weight cues

## Abstract

Recent research shows that the intention to act on an object alters its neural representation in ways as afforded by underlying sensorimotor processes. For example, the intention to grasp and pick up an object results in representations of the object's weight. But these representations become grasp-specific only immediately before object lift if weight information is relayed through object material. This feature triggers earlier representations regardless of intention probably because material–weight contingencies are overlearned. In contrast, recently learned weight cues should be recalled deliberately during grasp planning resulting in early grasp-specific representations. Here, we examined how action intentions affect the representation of newly acquired color–weight contingencies. We recorded electroencephalography while human participants grasped or reached for objects that varied in shape and density as indicated by their color. Multivariate analyses revealed a grasp-specific reactivation of color during planning that was mirrored in beta band. This suggests that task relevancy influences the representation of color such that previously encoded color–weight contingencies may be reactivated as required for grasping, mediated top-down via working memory. Grasp-specific representations of shape and color were also present in theta band, perhaps reflecting attentional activity. These results provide novel insights into the interplay between cognition and motor planning processes.

## Significance Statement

Recent research shows that the object feature weight, conveyed via highly overlearned material–weight contingencies, is more prominently represented within electrophysiological signals when people intend to grasp an object versus reach for it. However, such differences occur late, immediately before object lift. Our study is the first to show that newly learned color–weight contingencies yield early task-specific representations during action planning; we find that color/weight representations, forming 140–210 ms after object onset, reactivate ∼270 ms during grasp, but not reach planning, with the same pattern arising for beta band. These data suggest that the brain deliberately processes weight information depending on intention, arguably involving working memory representations. Our results highlight the interplay of cognition and motor planning.

## Introduction

“I suppose it is tempting, if the only tool you have is a hammer, to treat everything as if it were a nail.” While Maslow’s (1966) statement referred to the biasing influence of task sets on cognitions, task sets also bias sensory and sensorimotor processes. The way we see the world depends on how we intend to act (Craighero et al., 1999; Bekkering and Neggers, 2002; Fagioli et al., 2007; Shin et al., 2010). More specifically, action intentions such as grasping have been associated with altered spatial attention (Schiegg et al., 2003; Baldauf et al., 2006; Baldauf and Deubel, 2009; Brouwer et al., 2009) and action-specific activation of early visual areas (Gallivan et al., 2019), arguably through sensorimotor feedback signals from parietal cortex (Andersen and Cui, 2009; Cui, 2014).

Consistent with feedback signals activating early cortex, multivariate electroencephalography (EEG) studies have recently shown that human participants form early neural representations of the shape of an object ∼100–250 ms after it appears and that these representations re-occur after ∼250 ms (Guo et al., 2019), but only when the participants intend to grasp the object, not when they plan to simply reach for it (Lee et al., 2024). These reactivation patterns of shape representations suggest that grasp intentions trigger a detailed visual analysis of grasp-relevant object features to extract information about how to guide the grasping fingers to the object during movement planning.

Around the same time of planning, grasp-relevant information about the object's weight has been found to form neural representations, too (Lee et al., 2024). However, these representations were largely the same regardless of whether participants intended to grasp or reach for objects. Only during movement execution, shortly before participants lifted an object, grasp-specific representations were observed (Lee et al., 2024; also see Klein et al., 2023). This suggests that grasp-specific computations of object weight are confined to the respective grasping phase when the weight of an object becomes relevant. However, the onset of grasp-specific computations might depend on what kind of weight cues are available.

Stable weight cues probably are heavily overlearned because they are frequently and consistently encountered in daily life. For example, Lee et al. (2024) used objects that were made of wood or steel (also see Gallivan et al., 2014). These materials are so common in daily life that weight information might be automatically generated as soon as the material becomes visible—regardless of whether weight information is relevant for an intended action. In contrast, arbitrary cue–weight contingencies (Ameli et al., 2008) might not be recalled automatically because they are not overlearned. For example, arbitrary color–weight contingencies, learned within the short time span of an experiment (Li et al., 2009), should be recalled only when they are relevant for the intended action. Furthermore, given that these arbitrary contingencies require cognitive resources (Li et al., 2009; Baugh et al., 2016), the respective cognitive processes, arguably involving representations in working memory (Li et al., 2009; Gallivan et al., 2014; Guillery et al., 2017; Zhang et al., 2023), should arise in an action-specific manner.

It follows that specifically when people intend to grasp an object, transient color–weight cues should trigger memory queries about the contingency, probably as soon as an object becomes visible and the resulting grasp-specific representations of color might be modulated top-down by working memory (Lundqvist et al., 2011; Richter et al., 2018; for review, Miller et al., 2018). Thus, we expected that specifically for grasping EEG signals should carry information about object color, probably in the form of reactivation patterns as previously observed for shape information (Guo et al., 2021; Lee et al., 2024).

Indeed, this information might be contained in beta band oscillations which have previously been implicated in the top-down modulation of sensory processing and working memory maintenance (Lundqvist et al., 2011; Richter et al., 2018; Fiebelkorn and Kastner, 2019), as well as the reactivation of task-relevant working memory content (Spitzer et al., 2014; Wimmer et al., 2016; Spitzer and Haegens, 2017). Theta band might be another promising candidate given its association not only with working memory processes (Tesche and Karhu, 2000; Sauseng et al., 2010; Hsieh and Ranganath, 2014) but also goal-directed and feature-based attention (Liebe et al., 2012; Fellrath et al., 2016; Harris et al., 2017).

To test these predictions, in the current study we presented participants with 3D-printed objects of different colors signaling different densities and, thus, weight and asked them to grasp or to merely reach for them. Our aim was to this way elucidate the interactions of the sensorimotor processes underlying grasp actions and cognitions so as to explore the immense adaptability of behavior under the guidance of human thought.

## Materials and Methods

### Participants

Twenty undergraduate and graduate students (11 females; mean age, 22.6) gave their written and informed consent to participate in the experiment and were compensated $15/h for their time. All participants had normal or corrected-to-normal vision and were right-handed (Oldfield, 1971). All procedures were in accordance with the Human Participants Review Sub-Committee (approval ID: 37329 for the neural and cognitive mechanisms underlying predictions and attention) and with the Declaration of Helsinki involving human participants.

### Stimuli

**Figure 1.**
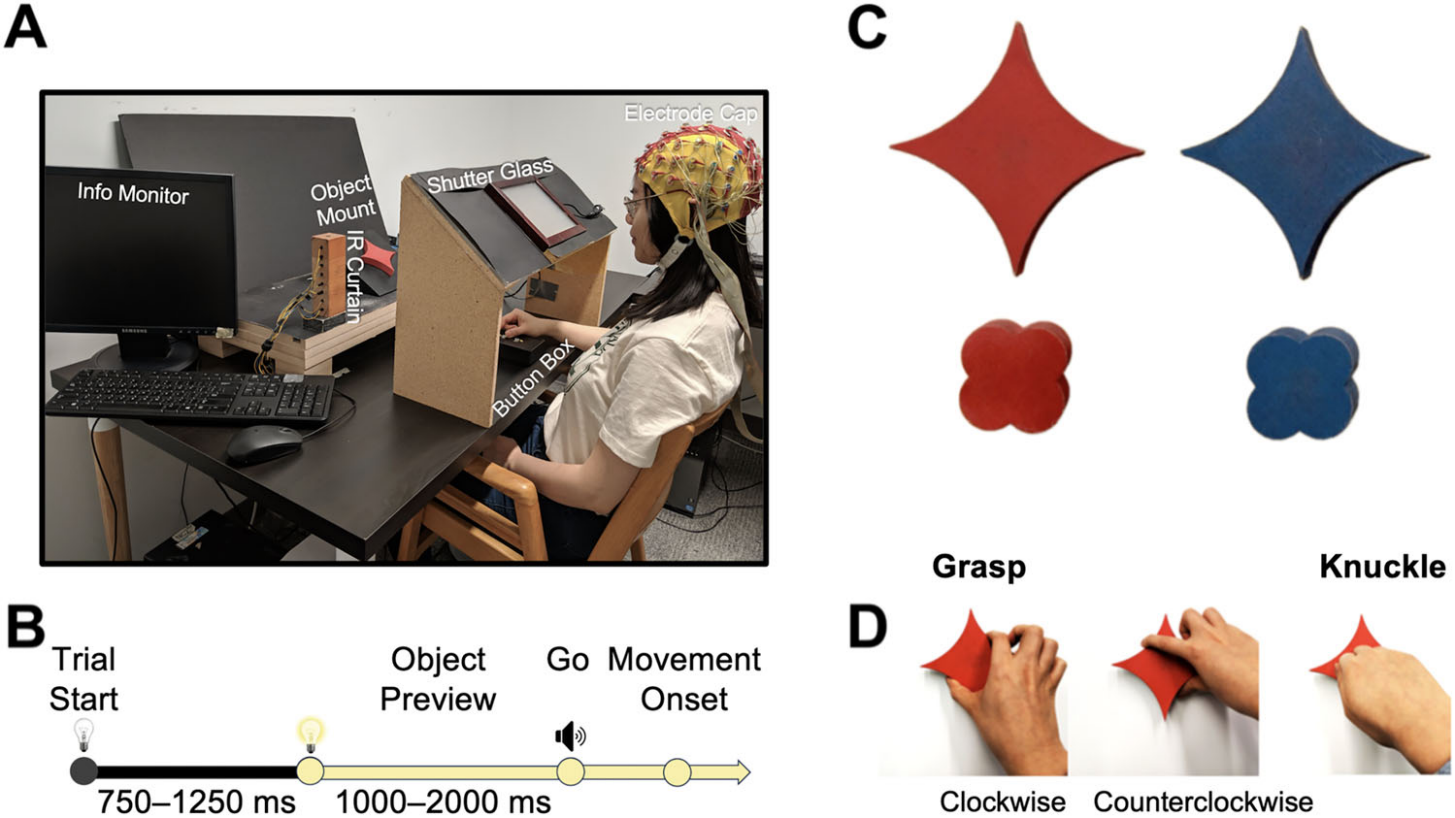
Experimental methods. ***A***, Experimental setup. ***B***, Timeline of a trial. ***C***, Objects used in the experiment. ***D***, Action conditions.

We introduced participants to four objects that differed in shape and color. Objects were blue and red 3D-printed “pillows” and “flowers” that were 2 cm in depth (Fig. 1*C*). Pillows had concave (72° segments of circles with a radius of 7.5 cm, i.e., curvature = 13.3/m), and flowers had convex sides (180° segments of circles with a radius of 1.5 cm, curvature = 66.7/m). These shapes were chosen to have visually distinct surface curvatures, while at the same time, all objects measured 6 cm across opposing edges, thus affording identical grip sizes. Critically, for a given participant, object sets (i.e., one pillow and one flower) of one color (i.e., red or blue, counterbalanced across participants) were hollow and thus 48% lighter compared with shapes of the opposing color (heavy pillow, 120 g; light pillow, 62 g; heavy flower, 63 g; light flower, 33 g). In this way, color was a reliable indicator of object density and weight. Thus, color, just like shape, was a visual feature that was relevant when we asked participants to grasp objects with their thumb and index finger, but not in the control condition where participants reached and touched the center of the objects with the knuckle of their index finger while making a fist (subsequently called “knuckling” in text).

### Procedure

Prior to the start of the experiment, participants were given a chance to lift each of the four objects. The experimenter explicitly drew attention to the fact the objects of one color were heavier than those of the other color. During each session, then participants sat in a dark room. The experimenter, seated on their left (Fig. 1*A*), prepared each trial; they viewed instructions on a monitor and turned on a pair of LEDs to mount objects on a platform in an otherwise dark grasp space. The platform was slanted toward the participant's line of sight, and a square-shaped hole in its center ensured that the objects, with a square peg attached to their back, were always placed in the same position and orientation, 43 cm away from the participant immediately behind a curtain of infrared beams (created by two 15-cm-tall pillars, 40 cm apart). Relative to the bottom of object, the infrared curtain was 6 cm away. This pretrial setup could not be monitored by the participant because they wore earplugs and had their view blocked by an opaque shutter glass screen (Smart Glass Technology). The participants then placed their right index finger and thumb on a button box, each covering a beam of infrared light to sense the presence of their hand. Next, the experimenter pressed a key that turned off the LEDs and, in darkness, set the shutter glass screen to transparent. Seven-hundred and fifty to 1,250 ms later, the LED lights turned on, thus illuminating the object for the participant to see for a “Preview” duration of 1–2 s before an auditory “Go” tone (loud enough to hear through the earplugs) signaled the participant to make their movement. As participants lifted their hand off the button box, this was recorded as movement onset, and the hand intersecting the infrared curtain was recorded as movement end. The kind of movement to be made (clockwise or counterclockwise precision grasps and lifts, or knuckles with a comfortable horizontal wrist orientation) was announced at the beginning of each block of trials (Fig. 1*D*) and monitored by the experimenter (they marked invalid trials when participants moved incorrectly or dropped objects; n.b., we included the variable Orientation merely to be able to compare to previous studies, Guo et al., 2019, 2021; Lee et al., 2024). There were 72 trials (2 shapes, 2 colors × 18 repetitions in random order) in one block, and there were 18 blocks in total (six for each movement type: clockwise grasps, counterclockwise grasps, and knuckles) administered in random order and in two sessions of ∼3 h each over two different days with breaks being provided between blocks as needed.

### EEG acquisition and preprocessing

We recorded EEG data using a 64-electrode BioSemi ActiveTwo recording system (BioSemi), digitized at a rate of 512 Hz with 24 bit A/D conversion. The electrodes were arranged according to the International 10/20 System, and the electrode offset was kept below 40 mV.

EEG preprocessing was performed offline in MATLAB using the EEGLAB (Delorme and Makeig, 2004) and ERPLAB toolboxes (Lopez-Calderon and Luck, 2014). Signals were bandpass filtered (noncausal Butterworth impulse response function, 12 dB/oct roll-off) with half-amplitude cutoffs at 0.1 and 40 Hz. Next, noisy electrodes which correlated <0.6 with nearby electrodes were interpolated (mean of 1.6 electrodes per subject), and all electrodes were rereferenced to the average of all electrodes. Independent component analysis (ICA) was then performed on continuous blocks for each participant to identify and remove blink and eye movement components (Jung et al., 2000; Chaumon et al., 2015; Drisdelle et al., 2017). The ICA-corrected data were segmented relative to the onset of Preview (−100 to 1,200 ms). In addition, invalid trials and epochs containing abnormal reaction times (<100 ms or >1,000 ms) were removed. As a result, an average of 4.1% of trials from each subject were removed from further analysis.

### Pattern classification of ERP signals across time

We averaged epochs into ERP traces to increase signal-to-noise ratio (SNR) of spatiotemporal patterns (Grootswagers et al., 2017). Specifically, we pooled together blocks of the same action (e.g., clockwise grasping). Up to 18 epochs within a given action block that corresponded to the same object (e.g., red pillow) were averaged together, resulting in six separate ERP traces per condition (e.g., clockwise grasping of red pillows) for Preview. We then performed multivariate noise normalization by calculating a covariance matrix for all time points of an epoch separately for each condition and then taking the mean of these covariance matrices across time points and conditions (Guggenmos et al., 2018). Next, we *z*-scored traces across time and electrodes, and outliers were thresholded at ±3 SD from the mean (for similar approaches, see Nemrodov et al., 2017, 2018). We opted for this winsorization approach to reduce the impact of outliers on support vector machine (SVM) pattern classification while maintaining the same number of features.

Further, we divided ERP traces into temporal windows with five consecutive bins (5 bins × 1.95 ms ≈ 10 ms) to increase the robustness of SVM analyses. For each time bin, data from all 64 electrodes were concatenated to create 320 features. In this way, we could perform time-resolved pattern classification across time through these bins.

For all decoding analyses, we first performed classification for each participant separately and then determined the mean performance across participants. Using SVM we conducted pairwise discrimination of Shape, Color, hand Orientation (on grasping trials only), and Action with a leave-one-out cross-validation approach (i.e., through iterations, all data were trained and tested on). More specifically, for Action, 35 of 36 pairs of observations were used in training, while one pair was used for testing. Due to the imbalanced nature of the number of grasping trials versus knuckling trials (i.e., there were twice as many grasping compared with knuckling trials), we set the cost parameter to be double the weight of the majority class (*c* = 2 vs *c* = 1; Batuwita and Palade, 2013) to avoid the classifier preferentially skewing toward the majority class. Further, we ensured the testing sample contained an equal number of minority (knuckling) and majority (grasping) instances. For the other independent variables, we separately analyzed grasping and knuckling trials. For Shape, Color, and Orientation, in the case of grasping: 23 of 24 pairs of observations were used for training and one pair was used for testing. For Shape and Color classification during knuckling: 11 of 12 pairs of observations were used for training and one pair was used for testing (there was not an equivalent Orientation condition for knuckling). For all Action and Orientation analyses, we created ERPs from randomly drawn blocks to account for blocked effects (due to action and hand orientation being held constant in a block, whereas objects varied by trial).

Further, to test for potential integrated representation of task features, we performed pairwise discrimination for Shape ∩ Color, separately for grasping and knuckling similar to Lee et al. (2024). More specifically, we trained and tested the SVMs with the following logic to reduce the possibility that classifiers relied only on one feature (i.e., shape or color) to classify: for the integrated representation of interest (e.g., “red pillow”) we included training and testing data that shared only the shape feature (e.g., “blue pillow”), only the color feature (e.g., “red flower”), or both features (e.g., “red pillow”). We treated this as an instance of imbalanced classes (see Action classification above) such that data containing both features would be considered the minority class, whereas data containing one feature would be considered the majority class. This resulted in a cross-validation setup of 33 observations used for training and two for testing for grasping and 15 observations for training and two for testing for knuckling. We trained four separate classifiers using this logic (i.e., the exhaustive combination of shape and color) and averaged their performance to obtain an estimate of Shape ∩ Color representations.

Next, we tested this averaged performance against an optimized additive effect between Shape and Color to determine whether these classifiers truly reflected integrated representations. To this end, we first calculated how a single-feature classifier would perform in our test:

$$p_{i}^{\prime }=0.75\times p_{i}+0.25\times (1-p_{i}),$$

where ***p_i_*** is the accuracy of some single-feature classifier ***i*** and ***p_i_***’ is its performance in our test (e.g., a shape classifier that attains an accuracy of *p*_shape_ = 0.7 would classify a minority object “red pillow” and a majority object “red flower” as belonging to class 1 and 2, respectively, with *p* = 0.7 each, and it would classify the second majority object “blue pillow” with *p* = 1 − 0.7 as belonging to class 2. This would result in correct classification with *p*_shape_’ = 0.5 × 0.7 + 0.25 × 0.7 + 0.25 × (1 − 0.7) = 0.75 × *p*_shape_ + 0.25 × (1 − *p*_shape_) = 0.6).

To then obtain an estimate of how two single-feature classifiers would perform optimally together, we modeled the response of each single-feature classifier as a continuous value with a Gaussian distribution where ***µ*** is the mean, ***σ_i_*** is the standard deviation, and ***p_i_***’ is the area under the Gaussian up to a decision boundary ***x***. The *p_i_*’ value can then be written as a cumulative Gaussian:

$$p_{i}^{\prime }=0.5\times (1+\mathrm{erf}(\left(x-\mu )/(\sigma_{i}\times \mathrm{sqrt}(2)\right)),$$

where **erf** is the error function and **sqrt** is the square-root. Solving Equation 2 for *σ_i_* for both single-feature classifiers (and with arbitrary values for *µ* and *x*) allows us to use maximum likelihood estimation to determine the optimally combined standard deviation for both single-feature classifiers together:

$$\sigma_{12}=\mathrm{sqrt}(\sigma_{1}^{2}\times \sigma_{2}^{2}/(\sigma_{1}^{2}+\sigma_{2}^{2})),$$

where ***σ*_1_** and ***σ*_2_** are the standard deviations for the two single-feature classifiers, respectively, and ***σ*_12_** is the standard deviation of the optimally combined classifier. Inserting ***σ*_12_** into Equation 2 gives us the optimal performance of both classifiers together (one exception: if one or two classifiers performed at guessing rate or worse, optimal performance *p*_12_ was set to the maximum of *p*_1_, *p*_2_, or 0.5). This way, the accuracy of the integrated classifier was tested relative to *p*_12_.

### Cross-temporal generalization of ERPs

We used cross-temporal generalization of ERPs to investigate whether classifiers trained on one time point can decode data from the same or different time points. Thus, we trained and tested classifiers on all combinations of times from the first 500 ms of Preview (Fig. 3). We shortened the time window as analyses beyond 500 ms did not yield significant results. To further examine the differences and similarities of how actions modulate representations, we conducted separate analyses: training and testing on grasping and training and testing on knuckling. If there were similarities in the representation of two selected time windows, the classifier should generalize between them. More specifically, there were three possible patterns of classifier performance we expected to see: (1) Chained representations. Represented by a classification pattern along the diagonal of the graph, the classifier should perform above chance only when trained and tested on the same time points. Transient neural representations that activate sequentially would likely result in this pattern. When seen in isolation, this would suggest the feature does not share representations across time and would be inconsistent with our hypothesis of encoded feature information that is used at later times. (2) Reactivated representations. Represented by significant classification along the diagonal as well as in approximately symmetrical “arms” above and below the diagonal. This means that the classifier trained at an earlier time performs above chance for later time points, reflecting that later neural representations are reactivated versions of earlier ones. We hypothesized to see this pattern of representation for Grasping and not Knuckling for Shape and Color, reflecting the requirement of earlier encoded feature information to successfully carry out the grasp. (3) Sustained representations. Represented by a wide coverage of areas above and below the diagonal, the classifier trained at an earlier time will perform above chance when tested for times immediately following the training time. Such a pattern of generalization would implicate a stable neural code or process over time. Although it would not necessary be inconsistent with our hypothesis, it would be unexpected based on past work to find this pattern for visual features (Guo et al., 2019, 2021; Guo and Niemeier, 2024; Lee et al., 2024). Instead, sustained representations have been found for highly robust features like action or grasp/reach orientation.

### Pattern classification of time frequency data

We performed classification of time frequency transformed data by first bandpass filtering (delta, 1–3 Hz; theta, 3–8 Hz; alpha, 8–12 Hz; beta, 15–25 Hz; gamma, 30–40 Hz) epochs using two-way least-squares finite impulse response filtering. We applied Hilbert transform on narrowband data to obtain the discrete-time analytical signal and squared complex values to obtain the power. This band-separated power was grouped, normalized, and averaged in the same way as ERP traces for spatiotemporal EEG classification (see above, Pattern classification of ERP signals across time and Cross-temporal generalization of ERPs).

### Electrode informativeness

We determined the informativeness of electrodes for the classification of ERP effects (Shape, Color, Shape ∩ Color, Orientation, and Action for data aligned to Preview) by conducting a searchlight analysis across electrodes. For each electrode we defined a 50-mm-radius neighborhood and performed classification on spatiotemporal features obtained from each electrode neighborhood across 100 ms time bins. For Shape ∩ Color, the values at each electrode were subtracted from the respective values obtained from the optimal integration of both classifiers together at each time bin and at each electrode (see above, Pattern classification of ERP signals across time).

### Significance testing

For behavioral data, we determined statistical differences (reaction and movement time) using three-way repeated-measures ANOVAs (Action × Shape × Color), adjusting for sphericity violations using Greenhouse–Geisser (GHG) corrections. We assigned Action as a three-level factor (i.e., clockwise grasping, counterclockwise grasping and knuckling) for completeness, but we did not find any difference between the two grasping conditions and subsequently did not treat these as separate groups for classification analyses. For post hoc analyses, we used repeated-measures *t* tests with Bonferroni’s correction for multiple comparisons.

For all analyses conducted on EEG data, we assessed statistical significance using a nonparametric, cluster-based approach to determine clusters of time points (or electrodes for searchlight analyses) where there were group-level significant effects (Nichols and Holmes, 2002). For example, in the case of time-resolved analyses, we defined clusters as consecutive time points that exceeded the 95th percentile of the distribution of *t* values at each time point attained using sign-permutation tests computed 10,000 times, equivalent to *p* < 0.05, one-tailed. Further, significant clusters were determined by cluster sizes equal to or exceeding the 95th percentile of maximum cluster sizes across all permutations (equivalent to *p* < 0.05, one-tailed). A similar approach was taken for searchlight analyses carried out across electrodes, and cluster-based correction was performed on each 100 ms time window, separately on spatial clusters of electrodes.

In the case of temporal generalization analyses, we tested for significant differences between grasping and knuckling for the three expected types of representations (i.e., chained, reactivated, and sustained representations), as well as a baseline (the first 50 ms, when representations are not yet expected in visual cortex) during Preview (Fig. 3). We obtained these regions of interest (ROIs) from independent criteria (see Lee et al., 2024, Significance testing for cross-temporal generalization), which will henceforth be referred to as “diagonal” ROIs (indicative of chains of multiple neural generators of the representations), “arms”-shaped ROIs (indicative of generators that reactivate at a later time), and “triangular” ROIs (indicative of generators that are active for a longer, sustained time; King and Dehaene, 2014).

For each of these ROIs, we obtained corresponding classification accuracy values from grasping and knuckling trials separately and then calculated a difference score of the average grasping versus knuckling accuracies at the respective ROIs. We then determined the one-tailed 95% confidence intervals of these differences (because a priori we expected representations during grasping to be more pronounced than representations, if any, during knuckling) using nonparametric bootstrapping with 10,000 iterations. For the diagonal, arms-shaped, or triangular ROI differences to be significant, these values had to be more positive than the difference for the baseline ROI with no overlap in confidence intervals to ensure that classification was better than based on unspecific differences between experimental conditions. We completed this significance testing process for all grasping versus knuckling comparisons of main effects and integrated representations. A significant difference within the diagonal ROI would suggest a stronger chained representation. A significant difference within the “arms”-shaped ROI (but not the triangular ROI) would suggest a stronger reactivated representation for grasping than knuckling. Furthermore, a significant difference within the triangular ROI (with or without differences in the arm-shaped ROI) would signify a stronger sustained representation for grasping compared with knuckling.

## Results

### Behavioral results

The mean reaction time (RT; defined as the time between Go onset and movement onset) was 286 ms (SD = 72 ms), and mean movement time (MT; defined as the time between movement onset and movement end) was 244 ms (SD = 49 ms). None of the main effects or interactions of the ANOVA for RT were significant (*F*'s < 1.798, *p*'s > 0.196). However, there was a main effect of Action for MT (*F*_(2,38)_ = 22.200, *p* < 0.01, *η_p_*^2^ = 0.539). Pairwise comparisons with Bonferroni’s correction revealed that knuckling trials had slower MTs (*M* = 272, SD = 41 ms) compared with both clockwise grasping (*M* = 226 ms; SD = 41 ms) and counterclockwise grasping (*M* = 235 ms; SD = 52 ms), but grasping conditions were not significantly different from one another. All other main effects and interactions were nonsignificant (*F*'s < 0.854, *p*'s > 0.367).

### Time-resolved classification of ERPs: investigating the time course of feature representations

**Figure 2.**
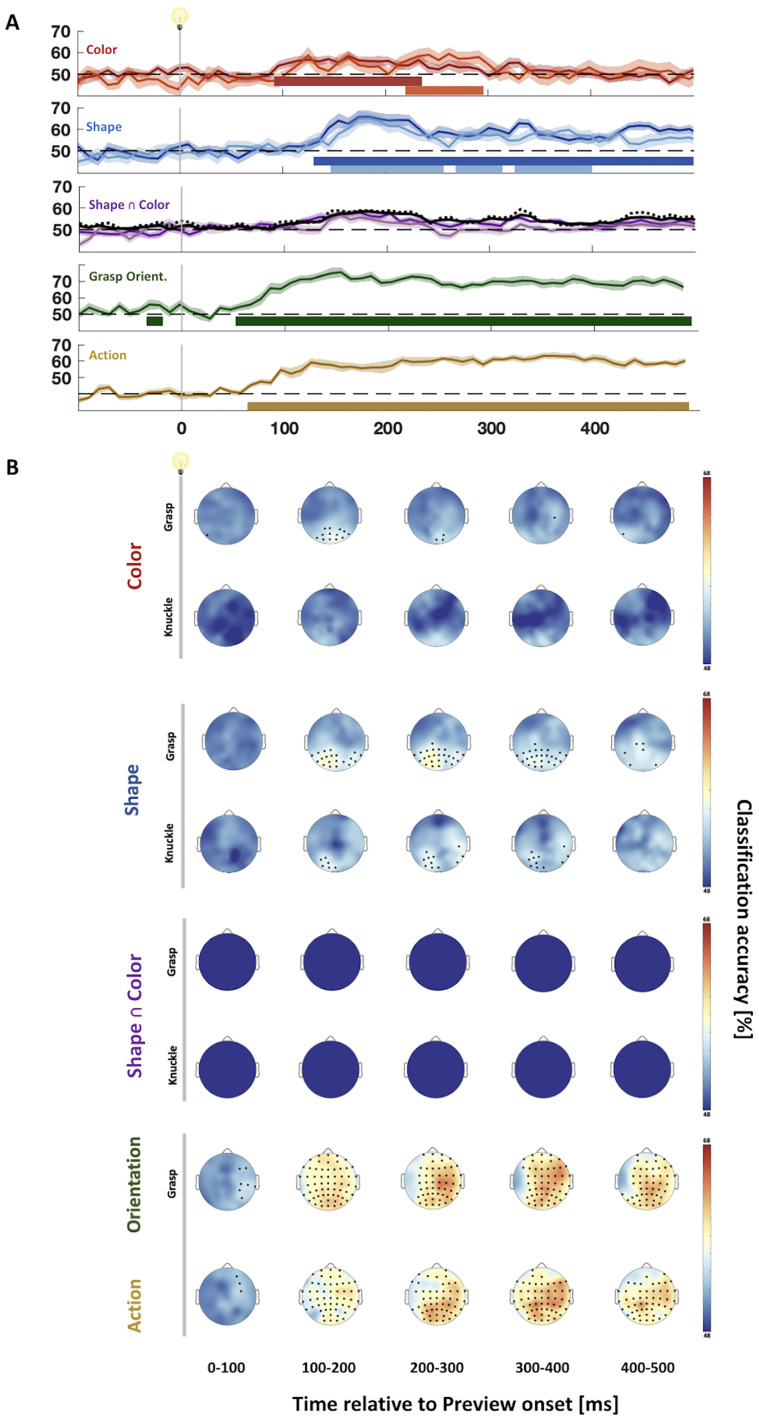
Classification accuracy of main effects and Shape ∩ Color during Preview. ***A***, Time-resolved classification of effects aligned to Preview onset. Darker curves represent classification of grasping data, and the lighter ones represent knuckling data. Black curves in the third graph show performance as predicted by the respective two single-feature classifiers for grasping (solid curves) and knuckling (dotted curves). Horizontal colored bars denote significantly above chance classification (cluster-corrected *t* tests, one-tailed; *p* < 0.05). ***B***, Electrode informativeness for respective effects aligned to Preview (two-tailed one-sample *t* test; *q* < 0.05). No Shape ∩ Color classifiers performed better than optimal additive models of single classifiers.

Time-resolved Grasping Color representations became significant from 90 to 230 ms, reaching a maximum at 160 ms (Fig. 2*A*, first row, dark red), whereas Knuckling Color representations were significant much later, from 220 to 290 ms and peaking at 260 ms (Fig. 2*A*, first row, light red), although there were no differences between Grasping and Knuckling.

There also were no significant differences between Grasping versus Knuckling Shapes, although both conditions were significantly different from chance. In detail, Grasping Shape representations became significant starting 130 ms after object onset, quickly rising to a peak at 180 ms and remaining significantly above chance for the rest of the Preview phase (Fig. 2*A*, second row, dark blue). In comparison, Knuckling Shape representations became significant slightly later than Grasping representations at 150 ms, though also reaching a peak at 180 ms. These Knuckling Shape representations were significant until 400 ms after Preview Onset (breaks at 250–270 ms and 310–330 ms; Fig. 2*A*, second row, light blue).

Addressing the question of whether there were integrated representations of Shape and Color, we tested classification accuracies of Shape ∩ Color against optimal additive models of single-feature classifiers (Shape and/or Color classifiers; Fig. 2*A*, third row; see Materials and Methods, Pattern classification of ERP signals across time for description of models). Neither Grasping Shape ∩ Color nor Knuckling Shape ∩ Color representations came out significant against the optimally integrated additive models, and moreover, there were no significant differences between grasping and knuckling trials.

Finally, merely to be comprehensive we also examined the two remaining experimental manipulations, Orientation and Action. We found that representations of Grasping Orientation (i.e., clockwise vs counterclockwise grasping; n.b., no Knuckling Orientation conditions were tested) were briefly significant before Preview Onset (−30 to −20 ms) and more substantially from 60 ms after Preview Onset, rising to a peak at 150 ms and remaining robustly significant until the end of the Preview period (cf. Guo et al., 2019, 2021; Lee et al., 2024).

Classification of Action (i.e., grasping vs knuckling) became significant at 70 ms, first rising quickly, then continuing to gradually rise, and becoming sustained until the end of Preview (highest classification accuracy was at 360 ms; Fig. 2*A*, fifth row; similar to Lee et al., 2024).

### Electrode informativeness of feature representations

To explore where information originated, we trained classifiers with a searchlight approach (individual electrode data plus their immediate vicinity). Grasping Color representations were significant at posterior electrodes starting from 100 to 200 ms (peak electrodes: Oz, O2, and PO4 which remained significant at 200–300 ms), implicating visual areas. No electrodes reached significance for Knuckling Color representations (Fig. 2*B*, first and second rows).

Grasping Shape information mostly originated from posterior electrodes, with peak classification lateralized to the left starting during the 100–200 ms interval and becoming most informative at 200–300 ms (peak electrodes including O1, PO3, and P3; Fig. 2*B*, third row), again implicating visual areas. Similarly, Knuckling Shape representations originated from posterior electrodes, with peak classification mostly on the left posterior side, though C6, TP8, and P8 also became significant between 200 and 400 ms (Fig. 2*B*, fourth row).

In the case of integrated Shape ∩ Color representations, no electrodes were significant after correcting for additive models (Fig. 2*B*, fifth and sixth rows).

Grasp Orientation information, in contrast, was widely dispersed, being most significant at posterior electrodes from 100 to 200 ms, lateralizing to the right from 200 to 300 ms (peak electrodes: C2, C4, and CP2) as well as 300–400 ms (peak electrodes: CP2, FC4, and FC6), and 400–500 ms (peak electrodes: C4, P2, and P2), suggesting a greater role of premotor and motor regions in the representation of orientation.

Action representation was also widely dispersed. From 100 to 200 ms, peak electrodes were lateralized to the right at frontocentral regions (including FC2, FC4, and FC6). Starting from 200 ms, there was greater involvement of posterior and centroparietal electrodes (from 200 to 300 ms peak electrodes on the left side, PO3 and P1, as well as the right side, CP4; from 300 to 400 ms peak electrodes also included the left and right sides, CP1, CP4, and F6; from 400 to 500 ms, CP1 featured most prominently), consistent with what would be expected of representations that would involve more premotor and motor regions.

### Temporal generalization of ERPs: testing for reactivation of feature representations

Central to the current study and informed by our previous work (Lee et al., 2024), we expected task-dependent differences between Grasping and Knuckling to arise from temporal generalization analysis. That is, we anticipated differences between these conditions in how the representations of experimental variables changed over time. We hypothesized to see grasp-specific reactivation of visual features. Further, Lee et al. (2024) did not find a reactivation of weight during action planning when this information was relayed through overlearned material cues. Here, we expected to see evidence that weight obtained from newly associated color cues will be represented during action planning as part of the maintenance of this weight information.

**Figure 3.**
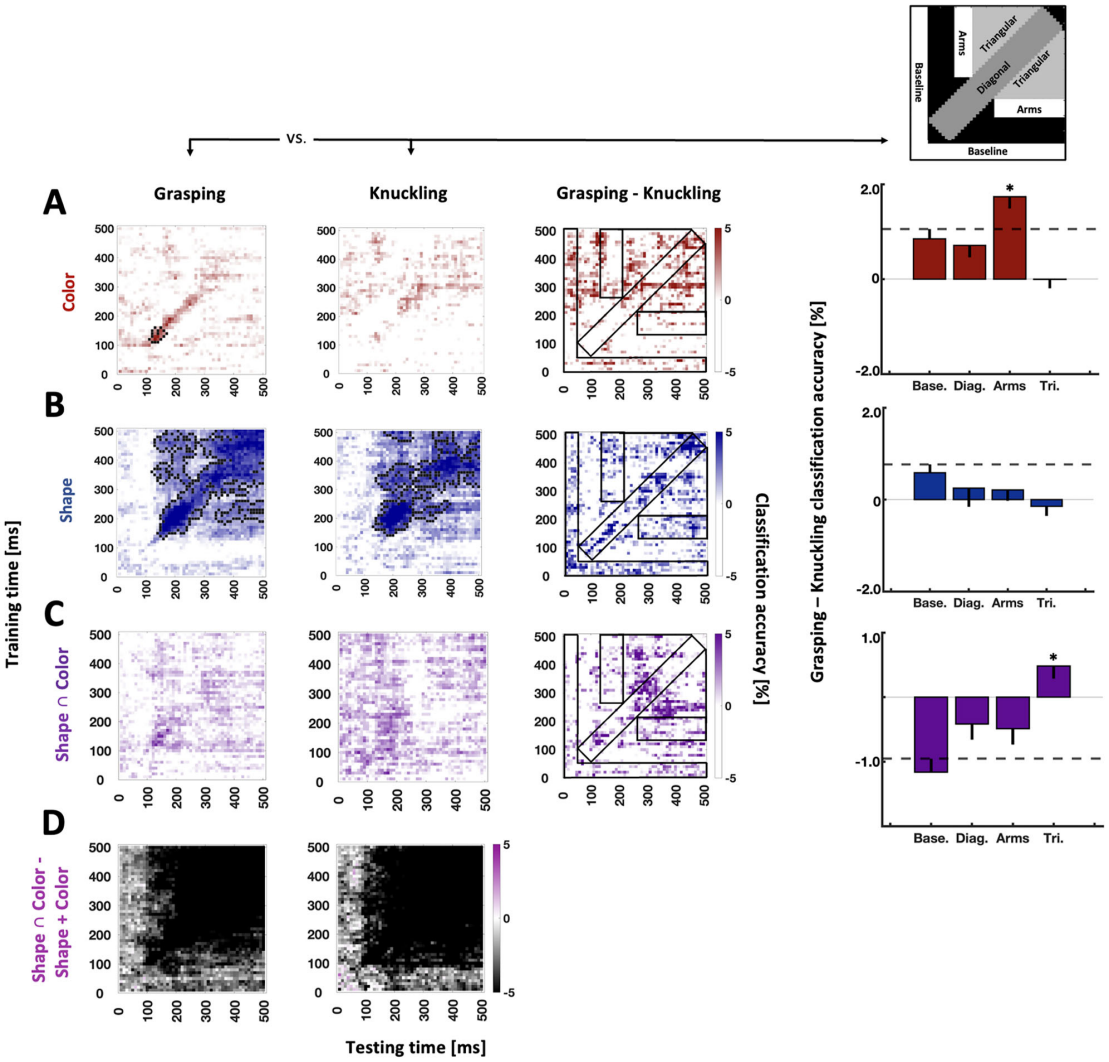
Temporal generalization of Color (***A***), Shape (***B***), Shape ∩ Color (***C***), and difference plots of Shape ∩ Color versus additive models of Shape and Color (***D***) aligned to Preview onset. Left side, Time-by-time plots for grasping (first plot), knuckling (second plot), and the difference of the grasping and knuckling plots (third plot). Black pixels in the time-by-time plots in columns 1 and 2 mark the contours of significant clusters (cluster-based sign-permutation test with cluster-defining and cluster-size thresholds of *p* < 0.01). Top right graph, ROIs as defined based on Lee et al. (2024). The same ROIs are superimposed as black outlined rectangles and L-shaped areas onto the plots in the third column. Right side, Bar graphs depict observed differences between grasping and knuckling for the ROIs. Base, baseline ROI; Diag, diagonal ROI; Arms, “arms”-shaped ROI; Tri, triangular ROI. Error bars represent bootstrapped one-tailed confidence intervals (10,000 iterations, one-tailed). Dashed lines visualize the upper boundary of the confidence interval for the baseline ROI. Asterisks represent significant results relative to baseline ROI.

Examining Grasping Color representations, we observed significance along the diagonal (from 110 to 170 ms along the *x*-axis and 110–160 ms along the *y*-axis in Fig. 3*A*, first graph) while no significance emerged for Knuckling Color (Fig. 3*A*, second graph). Crucially, comparing differences between Grasping versus Knuckling data for our a priori defined ROIs revealed that Color representations along the “arms”-shaped ROI were stronger for grasping than knuckling (Fig. 3*A*, third and fourth graph), suggesting that the intention to grasp an object (more so than the intention to knuckle an object) caused neural generators of color representations to reactivate.

Grasping Shapes analyses revealed significance relative to chance both off and along the diagonal of the classification matrix (Fig. 3*B*, first graph). Likewise, Knuckling Shapes representations were significant both along the diagonal and in noncontinuous regions off-diagonal (Fig. 3*B*, second graph). Comparing Grasping and Knuckling Shapes data with one another (Fig. 3*B*, third graph), we observed a nonsignificant trend for greater Grasping than Knuckling accuracy within the arms-shaped ROI. However, the trend remained below the threshold defined by the baseline ROI, and no other ROIs was significant either (Fig. 3*B*, fourth graph).

For Shape ∩ Color representations, we observed significantly greater accuracy for grasping than knuckling within the triangular-shaped ROIs (Fig. 3*C*, bar graph). However, this difference does not seem to amount to evidence for a truly integrated representation because, as shown in Figure 3*D*, at almost all times Shape ∩ Color representations were outperformed by additive models (i.e., nearly all difference values were negative).

**Figure 4.**
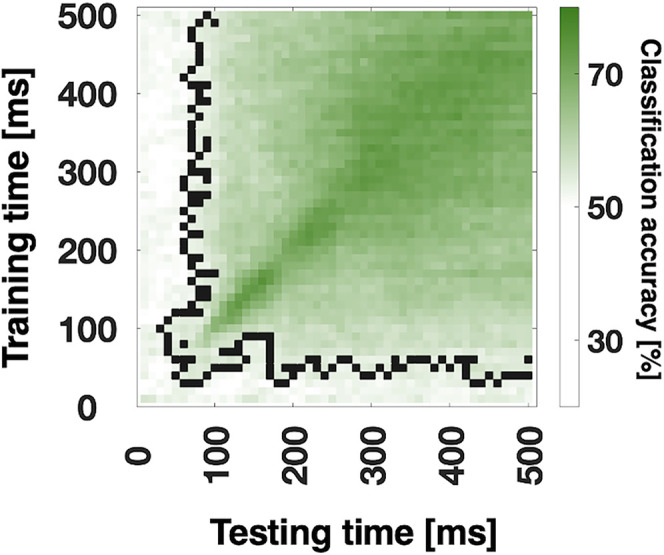
Temporal generalization of Grasp Orientation. Black pixels in the time-by-time plots in columns 1 and 2 mark the contours of significant clusters (cluster-based sign-permutation test with cluster-defining and cluster-size thresholds of *p* < 0.01).

We also performed analyses for Grasp Orientation (Fig. 4). Due to the nested design of our experimental conditions, the equivalent was not available for Knuckling. We found a robust sustained representation for Grasp Orientation starting as early as 40 ms to the end of the action planning period.

### Time frequency analyses: investigating oscillatory sources for grasp-specific reactivation

**Figure 5.**
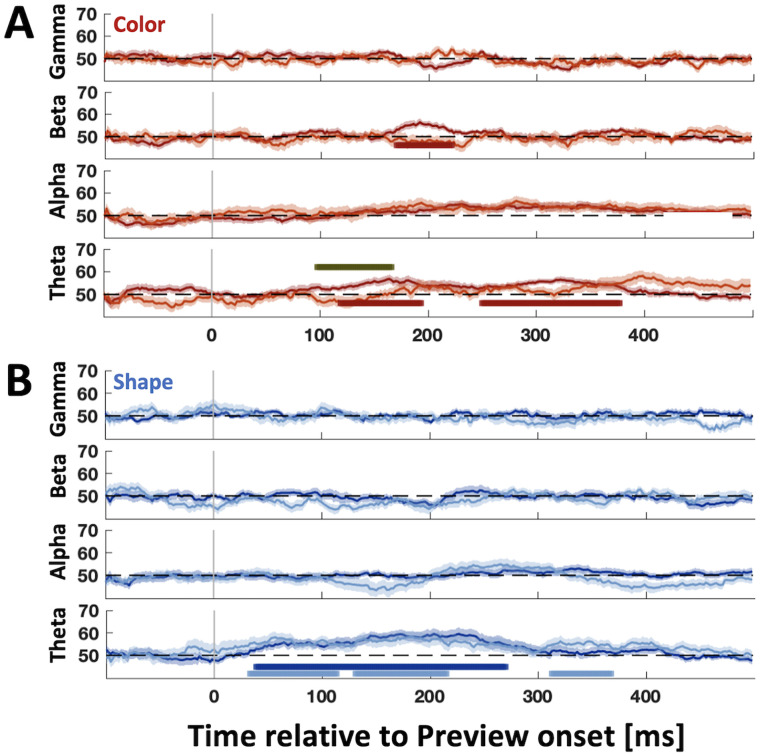
Time frequency analysis of Color and Shape representations aligned to Preview. Time-resolved plots of Shape and Color classification for time frequency data within separate frequency bands for Color (***A***) and Shape (***B***). Darker curves, grasping data; lighter curves, knuckling data. Horizontal colored bars represent above-chance classification accuracies (cluster-corrected *t* test, one-tailed; *p* < 0.05). Gray horizontal bars denote significantly better classification for grasping compared with knuckling (cluster-corrected *t* test, one-tailed; *p* < 0.05).

To further investigate the potential oscillatory sources of our two main grasp-relevant variables, Shape and Color, we conducted classification for separate frequency bands. In terms of time-resolved analysis of Color (Fig. 5*A*), we found significance in the beta band for Grasping Color from 170 to 220 ms (maximum at 190 ms). Theta band yielded significant Grasping Color representations from 120 to 195 ms and from 250 to 375 ms (maximum at 160 ms), with Knuckling Color representations remaining nonsignificant. However, the difference between Grasping and Knuckling Color was significant between 95 and 165 ms. There were no significant representations for alpha or gamma.

For Shape (Fig. 5*B*), Grasping Shape representations were significant for theta between 40 and 270 ms (maximum at 200 ms), whereas Knuckling Shape was significant from 30 to 220 ms except for an interruption from 110 to 130 ms (maximum at 180 ms), as well as significant from 310 to 370 ms. Grasping and Knuckling Shape curves were statistically not different from one another. No other frequency band yielded significant shape effects.

Next, given the significant time frequency classification results from Figure 5 as criterion to also analyze their temporal dynamics, we performed temporal generalization analysis for Color within theta and beta band and for Shape within theta band to look for differences between Grasping and Knuckling.

**Figure 6.**
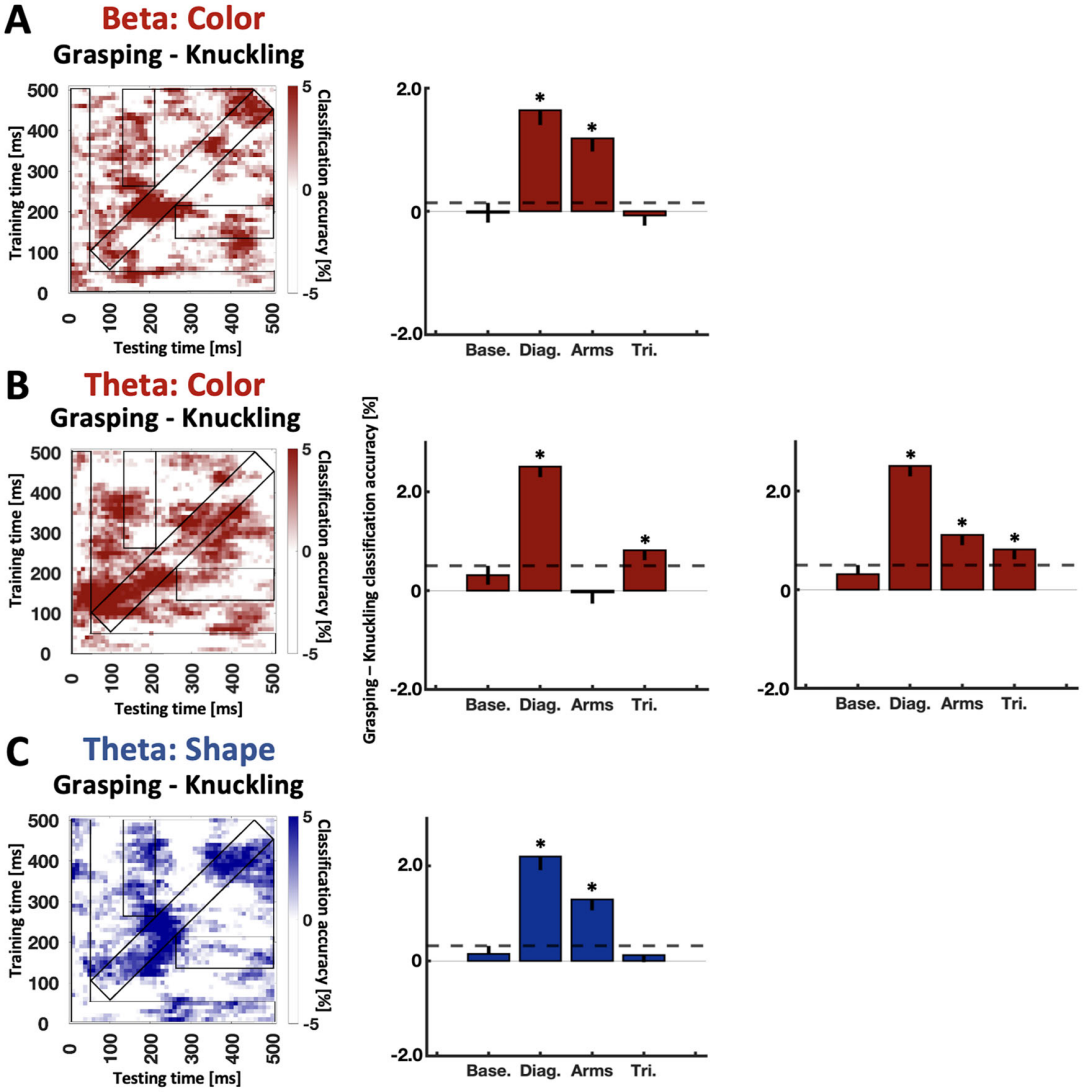
Temporal generalization of time frequency data for Grasping versus Knuckling differences for Color and Shape representations aligned to Preview. ***A***, Color classification for beta, (***B***) Color classification for theta, and (***C***) Shape classification for theta. The outlines of the ROIs used to calculate classification differences between grasping and knuckling have been superimposed in black. Bar graphs depict differences between grasping and knuckling for the predefined ROIs based on Figure 3. Rightmost bar graph depicts differences between grasping and knuckling for theta color classification when “arms”-shaped ROIs are shifted 50 ms earlier (90–160 ms). Base, baseline ROI; Diag, diagonal ROI; Arms, “arms”-shaped ROI; Tri, triangular ROI. Error bars represent bootstrapped confidence intervals (10,000 iterations, one-tailed). Horizontal dashed lines visualize significance level (i.e., the upper boundary of the confidence interval for the baseline ROI). Asterisks represent significant results relative to baseline ROI.

**Figure 7.**
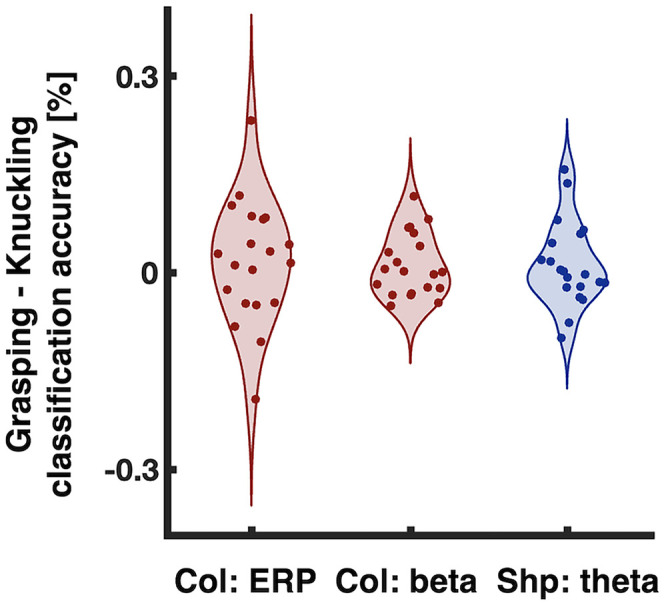
Participant difference scores between Grasping and Knuckling classification accuracies for “arms”-shaped ROIs for (from left to right): Col: ERP, Color classifier trained on ERP data; Col: beta, Color classifier trained on beta band data; Shp: theta, Shape classifier trained on theta band data.

For Color we observed that Grasping versus Knuckling differences within beta band produced better classification along the diagonal and the “arms”-shaped ROI, consistent with reactivation of representations (Fig. 6*A*). In addition, classifying within theta band yielded a significant effect along the diagonal and in the “triangular” regions of interest, consistent with sustained representations (Fig. 6*B*, second plot). Theta band produced no significant effect for temporal boundaries of the “arms”-shaped ROI as defined by our previous study (Lee et al., 2024). However, for exploratory reasons we also inspected an “arms”-shaped ROI for an earlier time (90–160 ms) given the grasp-specific difference observed for the time-resolved analysis (Fig. 5*A*, theta band). For this post hoc defined ROI, we did observe significantly better classification for Grasping Color compared with Knuckling Color (Fig. 6*B*, third plot). Further testing is required to confirm whether the difference reflects earlier reactivation of grasp-specific Color representations within theta band. Lastly, Grasping Shapes versus Knuckling Shapes within theta band yielded significantly better classification along the diagonal and “arms”-shaped ROIs, consistent with what would be expected of a reactivation pattern (Fig. 6*C*). For a breakdown of individual decoding accuracy differences between Grasping and Knuckling for “arms-shaped” ROIs, refer to Figure 7.

## Discussion

In the present study we tested how the intention to grasp an object influences cortical representations of arbitrary weight cues that are transiently stored in memory. Human participants first learned that red and blue colored objects were made from solid and hollow blocks of plastic, respectively, or vice versa and then grasped the objects while we used EEG to record their brain activity. Training classifiers on the data, we found that representations of object color generalized in time more strongly when participants planned to grasp the objects compared with when they intended to touch them with their knuckle.

### Cognitive mechanisms implicated in reactivated color representations during grasp planning

In detail, the task-specific difference arose within “arms-shaped” ROIs of the temporal generalization plot (Lee et al., 2024), a pattern that is indicative of earlier representations being reactivated at later times (King and Dehaene, 2014). Furthermore, we found the same reactivation pattern within beta band. Our results suggest that the colors of the objects were represented as action-specific cues in working memory in the context of motor planning extracting information about the weight of objects.

The current findings differ from previous results where representations of object material (and therefore possibly representations of weight information) occurred during similar times but were not grasp-specific (Lee et al., 2024). In the previous study, weight cues came from materials that are commonly used in daily life with overlearned material–weight contingencies. Therefore, weight information was likely recalled automatically when objects became visible, no matter what participants intended to do. In contrast, the color–weight cues in the present study were based on newly stored contingencies. Therefore, contingencies would not have been automatically recalled—or at least such recall would have been much less probable. Instead, recall should have happened mostly, if not exclusively, when participants viewed the objects with the intention to grasp them. Consistent with this reasoning, we observed reactivation of color representations within ERPs when participants were planning to grasp objects.

What is more, the intention-triggered memory query about the respective color–weight contingency should then have been represented in working memory. Maintaining such working memory content and top-down influences of working memory on sensory inputs have been shown to be prominently implemented within beta band oscillations (Engel and Fries, 2010; Lundqvist et al., 2011; Richter et al., 2018; Fiebelkorn and Kastner, 2019). Indeed, here we found that classifiers trained on beta band revealed the same grasp-specific reactivation pattern of color as found for ERPs. In sum, this suggests that action intention triggering the recall of the color–weight contingency resulted in a working memory-guided reactivation of neural processing of color after 270 ms that recapitulated earlier color processing from 140 to 210 ms, presumably to convert color cue into weight information. This could be consistent with observations from delayed match-to-sample tasks that have shown that beta modulations are correlated with successful discrimination and are found later in the delay (Spitzer et al., 2010; Wimmer et al., 2016; Spitzer and Haegens, 2017) when working memory information might be reactivated for the impending comparison task. An alternate explanation might be that information contained in beta is instead linked to the maintenance of the sensorimotor set (Androulidakis et al., 2006; Pogosyan et al., 2009; Swann et al., 2009) or reflects anticipatory motor processes (Schoffelen et al., 2005; Donner et al., 2009), perhaps related to the expected sensory inputs of the color cue. However, we will note that our previous work using a similar paradigm except with objects made of common materials did not yield grasp-specific material representations until the time of object load (Lee et al., 2024). Future work will have to disentangle the extent these processes play for representing newly learned weight associations in beta band.

Preceding the reactivation within beta band, a grasp-specific color representations also emerged within theta band from 95 to 165 ms after object onset. The effect could mark an early onset of weight computations. But more plausible is that it reflects a shift of object- or feature-based attention to the object and its color (Liebe et al., 2012; Harris et al., 2017). We found that these theta band representations were significant along the diagonal of the temporal generalization plot suggesting that the representations resulted from a chain of neural generators representing (King and Dehaene, 2014) whereas our predefined “arms-shaped” ROIs (Lee et al., 2024) returned no evidence for grasp-specific reactivation. That said, it is reasonable to assume that participants shifted attention to the object's color before recalling the color–weight contingency (consistent with our observation that the time-resolved grasp-specific effect within theta band in Fig. 4*A* occurred earlier than the temporal boundaries of the “arms-shaped” ROIs). Thus, the predefined “arms-shaped” ROIs might not have been suitable to identify an effect of reactivation. Further research is required to confirm whether the earlier ROIs as chosen post hoc in the current study continue to yield a grasp-specific reactivation effect within theta band.

Another form of action-specific reactivation was observed for classifying shape. This was similar to previous studies (Guo et al., 2019; Lee et al., 2024) although in the present study, reactivation occurred within theta band rather than for ERPs. This disparity might simply be due to natural fluctuation in the EEG data. Alternatively, we speculate that it might indicate that here reactivation of shape was different or perhaps weaker due to greater cognitive demands of the color–weight contingency. As another possibility, the current paradigm might have afforded feature-based attention to shift away from the shape of the object to its color. This would be consistent with the proposed role of theta in mediating attentional shifts, as well as enhancing target representations during encoding and retention (de Vries et al., 2019; Riddle et al., 2020; Magosso and Borra, 2024; Ohgomori et al., 2025). A third possibility might be that the timing in the current study was different from before, i.e., present participants viewed objects for 1–2 s whereas in Guo et al. (2019) presentation time was only 200 ms. On the other hand, presentation time in Lee et al. (2024) was 0.5–1 s and, thus, not considerably different. In addition, it is unclear why a longer presentation time should result in less pronounced shape representations. Next, it is difficult to argue that motor demands were different, as the shape, size, and distance of objects were virtually the same as before, and although there were differences in weight (only the present study used objects made from plastic), previous studies either used wooden objects that were lighter (Guo et al., 2019; Lee et al., 2024) or metal objects that were heavier (Lee et al., 2024). Finally, visibility of the present objects might have been slightly reduced but then, we did not observe substantial reductions in time-resolved shape classification accuracy (Fig. 2) compared with our earlier studies. Once again, further research is required to examine the influence of these processes on the pattern of reactivation observed.

In contrast, the present study is largely consistent with our previous one (Lee et al., 2024) in that we found little evidence for integrated representations of object features shape and surface properties (color vs material), consistent with our view that computations of shape (to program movement trajectories) and weight (to program object lifting) are separate from one another (Lee et al., 2024).

In conclusion, the present study offers novel insights into interactions of cognitive and action planning processes. We show that the processing of arbitrary weight cues for later grip force computations occurs in a grasp-specific manner, arguably guided by top-down signals from working memory. These findings are consistent with a model that grasp-specific computations occur upon the computational demand of the respective action phase (Guo and Niemeier, 2024; Lee et al., 2024). The fact that reactivation of visual information occurs for multiple object features (shape, color, as well as material) and at different times (object preview/movement planning vs load phase, see Lee et al., 2024) seems to indicate that reactivation of earlier neural processes is a general signature of action-guided enhanced visual processing.

## Synthesis

Reviewing Editor: David Franklin, Technische Universitat Munchen Decisions are customarily a result of the Reviewing Editor and the peer reviewers coming together and discussing their recommendations until a consensus is reached. When revisions are invited, a fact-based synthesis statement explaining their decision and outlining what is needed to prepare a revision will be listed below. The following reviewer(s) agreed to reveal their identity: Bianca Baltaretu.

Your manuscript has now been reviewed and discussed by the two reviewers and me. The reviewers have highlighted the strong methodology and results. While both reviewers appreciate the scientific study and potential advance in our understanding of how object properties are used to guide the planning and execution processes, we have some suggestions regarding the current manuscript. I particularly highlight some of the concerns regarding other possible interpretations of the data which should be discussed. The combined comments from both reviewers are found below. Please revise the manuscript taking these suggestions into account.

Summary and Comments from the Reviewers:

This study investigates how action intentions influence the neural representation of newly acquired color-weight contingencies during motor planning. The authors show that grasping (but not knuckling) triggers reactivation of color representations in ERP and beta-band signals, suggesting a role for working memory. Theta-band activity is also observed for grasp-specific shape and color representations, possibly reflecting attentional modulation. The findings contribute to our understanding of how cognition interacts with motor planning, particularly in the context of learned associations.

This study presents novel findings on how action intentions influence neural representations of learned weight cues. The methodology is strong, and the results are well-supported. However, certain interpretations-particularly regarding beta-band activity and shape representations-require additional clarification. This manuscript will make a valuable contribution to research on interplay between motor planning and cognitive processes.

1) The authors argue that beta-band reactivation reflects working memory involvement in retrieving color-weight associations. While this is a reasonable interpretation, beta-band activity has also been linked to sensorimotor maintenance and predictive coding (Engel &Fries, 2010). The authors should clarify how their findings distinguish between working memory-related reactivation and alternative explanations, such as anticipatory motor processes. If relevant, they could discuss connectivity analyses or cite studies that directly link beta-band activity to working memory in grasp-related tasks.

Engel AK, Fries P. Beta-band oscillations--signalling the status quo? Curr Opin Neurobiol. 2010 Apr;20(2):156-65. doi: 10.1016/j.conb.2010.02.015. Epub 2010 Mar 30. PMID: 20359884.

2) The study finds reactivation of shape representations in theta-band signals, whereas previous work (Lee et al., 2024) observed reactivation in ERP traces. The authors suggest that this difference may be due to increased cognitive demands in the current experiment. This explanation is speculative and not tested directly. Alternative explanations (e.g., differences in experimental timing, motor preparation demands?) should be considered. The authors should discuss why theta-band reactivation, rather than ERPs, was observed for shape. Could it be due to attentional shifts rather than memory retrieval? A comparison with previous studies would strengthen the discussion.

3) EEG decoding results rely on averaging across participants, but inter-individual differences in learning and memory retrieval could influence classification accuracy. Did all participants show grasp-specific reactivation, or was there substantial variability? A supplemental analysis showing individual decoding accuracies would help determine whether effects are consistent across the sample or driven by a subset of participants.

4) The authors should include a rationale in the introduction explaining the importance of frequency analysis in their study. Specifically, they should discuss how neural oscillations in the theta and beta bands are related to cognitive and motor processes such as motor planning, working memory, and attention. This will help clarify the value of frequency-domain analysis in understanding the neural mechanisms underlying action intentions and learned associations.

5) rename subsection titles in the Analysis - Discussion sections, and make explicit which hypothesis or questions the authors are addressing in each subsection.

6) given that there is no explicit testing of orientation (apart from consistency with the previous study), I would encourage the authors to add a supplementary section where this feature is discussed.

Minor comments:

1Where was the infrared curtain relative to the object being interacted with?

The labels in the set-up figure could be written in larger font.

Please discuss more explicitly each of the representations in the 'Cross-Temporal Generalization of ERPs' with respect to specific hypotheses/analyses, so as to clarify why they are being tested.

Please include, wherever appropriate (e.g., in the 'Electrode Informativeness' section), information about electrode and likely related cortical structure.
